# Acute stress selectively reduces reward sensitivity

**DOI:** 10.3389/fnhum.2013.00133

**Published:** 2013-04-11

**Authors:** Lisa H. Berghorst, Ryan Bogdan, Michael J. Frank, Diego A. Pizzagalli

**Affiliations:** ^1^Department of Psychology, Harvard UniversityCambridge, MA, USA; ^2^Center for Depression, Anxiety and Stress Research, Harvard Medical School, McLean HospitalBelmont, MA, USA; ^3^BRAIN Laboratory, Department of Psychology, Washington University in St. LouisSt. Louis, MO, USA; ^4^Departments of Psychiatry and Cognitive, Linguistic, and Psychological Sciences, Brown Institute for Brain Science, Brown UniversityProvidence, RI, USA

**Keywords:** affect-cognition interactions, stress, anhedonia, reward, punishment, cortisol, depression, emotion

## Abstract

Stress may promote the onset of psychopathology by disrupting reward processing. However, the extent to which stress impairs reward processing, rather than incentive processing more generally, is unclear. To evaluate the specificity of stress-induced reward processing disruption, 100 psychiatrically healthy females were administered a probabilistic stimulus selection task (PSST) that enabled comparison of sensitivity to reward-driven (Go) and punishment-driven (NoGo) learning under either “no stress” or “stress” (threat-of-shock) conditions. Cortisol samples and self-report measures were collected. Contrary to hypotheses, the groups did not differ significantly in task performance or cortisol reactivity. However, further analyses focusing only on individuals under “stress” who were high responders with regard to both cortisol reactivity and self-reported negative affect revealed reduced reward sensitivity relative to individuals tested in the “no stress” condition; importantly, these deficits were reward-specific. Overall, findings provide preliminary evidence that stress-reactive individuals show diminished sensitivity to reward, but not punishment, under stress. While such results highlight the possibility that stress-induced anhedonia might be an important mechanism linking stress to affective disorders, future studies are necessary to confirm this conjecture.

## Introduction

Unraveling the connection between life stress and the onset of affective disorders continues to be a critical but complex endeavor. The reward system is often dysfunctional in affective disorders (American Psychiatric Association, 2000) and may play a central role in bridging these phenomena. Specifically, mounting evidence suggests that stress attenuates reward responsiveness through its influence on underlying neurobiological processes (Anisman and Matheson, 2005). However, a central point of ambiguity in this domain concerns the specificity of the impact of stress on reward processing. In order to gain a more comprehensive understanding of the mechanisms at play, it is necessary to clarify whether such effects might be generalizable to other valence-laden stimuli (e.g., punishment) and thus reflective of incentive processing more broadly.

A large body of preclinical work suggests that uncontrollable negative stressors blunt sensitivity to reward via disruption of mesocorticolimbic pathways. The majority of research investigating relationships between stressors and reward processing has been performed in non-human animal studies. In rodents, uncontrollable stress leads to “anhedonic” behavior and dysfunction within mesocorticolimbic dopaminergic pathways critically implicated in incentive motivation and hedonic coding (Anisman and Matheson, 2005; Henn and Vollmayr, 2005). Surprisingly, relatively few researchers have empirically examined putative relationships between stress and the reward system in humans. In an early human study, Berenbaum and Connelly (1993) found that real-life acute stressors, including military training and final examinations, reduced self-reported pleasure and positive affect in two separate samples. Moreover, this stress-induced reduction in hedonic capacity was strongest in participants with family histories of depression. In a controlled laboratory setting, Bogdan and Pizzagalli (2006) reported that an acute stressor (threat-of-shock) blunted reward responsiveness—specifically, participants' ability to modulate behavior as a function of rewards (see Bogdan et al., 2011 and Liu et al., 2011 for independent replications). Using the same probabilistic reward task, participants with high levels of perceived life stress were characterized by decreased reward responsiveness (Pizzagalli et al., 2007). Recently, Cavanagh and colleagues (2010) employed a social evaluative stress manipulation while participants completed a probabilistic stimulus selection task (PSST). They found that stress led to relatively decreased reward learning in individuals with high trait-level punishment sensitivity [as assessed using the Behavioral Inhibition System (BIS) scale] as compared to an enhanced reward learning bias in individuals with lower trait-level punishment sensitivity. Complementing these behavioral findings, two recent neuroimaging studies reported that stress inductions (e.g., cold pressor task, aversive movie clips) superimposed on reward processing paradigms reduced activity in brain areas involved in reward processing, including the medial prefrontal cortex, orbitofrontal cortex, and dorsal striatum (Ossewaarde et al., 2011; Porcelli et al., 2012).

In spite of these findings, it remains unclear whether such stress-induced effects are specific to rewards or extend to negatively-valenced stimuli, such as punishment. In Cavanagh's aforementioned study (2010), social evaluative stress led to heightened sensitivity to punishment in individuals with high trait-level punishment sensitivity, but lower sensitivity to punishment in individuals with low trait-level punishment sensitivity. In related research, various prior studies have examined aversive processing changes using threat of shock manipulations and report stress-induced increases in aversive processing during affective Stroop tasks (e.g., Edwards et al., 2006, 2010; Robinson et al., 2011). In a recent fMRI study investigating the neural circuitry underlying such findings, Robinson and colleagues (2012) reported that enhanced dorsomedial prefrontal cortex amygdala connectivity during the processing of aversive stimuli under stress (threat of unpredictable foot shock in the scanner) might underlie stress-induced threat biases. Collectively, these studies raise the possibility that, unlike reward sensitivity, punishment sensitivity might be potentiated under stress.

The current study was designed to assess the specificity of the deleterious effect of stress on reward processing by comparing the impact of stress on reward-related (e.g., positive feedback) vs. punishment-related (e.g., negative feedback) learning. To achieve this aim, a PSST (modified from Frank et al., 2004) was implemented in conjunction with an acute stressor (threat-of-shock) using a between-subjects design (e.g., “stress” vs. “no-stress”). The current study design differed from previous studies in this area (e.g., Bogdan and Pizzagalli, 2006; Bogdan et al., 2011) because it allowed evaluation of responsiveness to both positive and negative feedback. This enabled us to ascertain whether purported stress-induced reward processing deficits reflected specific reductions in sensitivity to reward feedback vs. broad reductions in sensitivity to feedback in general (regardless of valence). In addition, our experiment was initially designed to test whether the impact of stress on reward processing was conditional upon the stressor being perceived as uncontrollable. This was attempted by implementing both a “controllable” and “uncontrollable” stress condition, along with a “no stress” condition. However, this aspect of our stress manipulation was unsuccessful (see Appendix for detailed analyses) and thus the present report focuses on the comparison between “stress” (collapsed across the two controllability subgroups) and “no-stress” conditions. Based on prior findings, we hypothesized that individuals under acute stress would exhibit reduced reward sensitivity (e.g., lower reward-related accuracy and a reduced reward-related RT bias, as detailed in the Materials and Methods section) relative to individuals in the no-stress condition. Moreover, we hypothesized that reward sensitivity would be selectively more reduced relative to punishment sensitivity in those individuals completing the task under stress.

## Materials and methods

### Participants

All study procedures were approved by Harvard University's Committee on the Use of Human Subjects in Research. One hundred (*n* = 100) female participants, 18–25 years old, were recruited through community advertisements and the Harvard University Department of Psychology Study Pool. Only females were recruited due to sex differences in psychological and hormonal responses to stress, and because women tend to demonstrate a more pronounced stress response than men (Nolen-Hoeksema and Hilt, 2009). All subjects were right-handed, non-smokers, with normal or corrected-to-normal vision, no color-blindness, and no known current or past neurological, psychiatric or medical illnesses. Prior to participation, all individuals were screened over the phone to determine study eligibility. The evaluation included diagnostic screening questions from the Structured Clinical Interview for DSM-IV Axis I Disorders (SCID; First et al., 1995), more detailed questions from the depression and substance abuse modules, and a handedness questionnaire (Chapman and Chapman, 1987). Subjects were excluded if they could speak or read Japanese because one of the tasks (PSST) included Hiragana symbols. Individuals who met eligibility requirements were invited for an experimental session. Prior to the session, participants were randomized to one of three experimental conditions: “no stress” (*n* = 29), “controllable stress” (*n* = 35), or “uncontrollable stress” (*n* = 36). Data from five participants (two from the “no stress” group, one from the “controllable stress” group and two from the “uncontrollable stress” group) were excluded because they never met performance criteria [see Modified Probabilistic Stimulus Selection Task (PSST) section] in the training phase of the PSST. Thus, 95 participants were included in the analyses: “no stress” group (*n* = 27), “controllable stress” group (*n* = 34), and “uncontrollable stress” group (*n* = 34). However, given the lack of success of the controllability aspect of our stress manipulation (see Appendix for detailed analyses), data from the two stress groups were combined into a single “stress” group in subsequent analyses.

### Procedures

**Figure 1:**
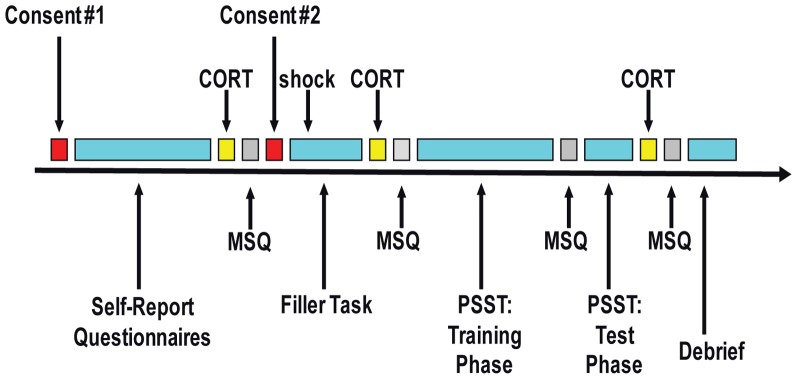
**Schematic representation of the session timeline.** CORT, collection of saliva sample to measure cortisol level; MSQ, mood state questionnaires (“in-the-moment” state self-report questionnaires); PSST, Probabilistic Stimulus Selection Task.

Figure 1 presents a summary of the session timeline. After arriving to the laboratory, the first written informed consent was obtained using a general consent form with no mention of the stress manipulation. This procedure allowed us to obtain unbiased baseline self-report ratings and physiological indices. Participants were then asked to complete a battery of self-report questionnaires, including a demographics form, the Beck Depression Inventory-II (BDI-II; Beck et al., 1996), the Mood and Anxiety Symptom Questionnaire (MASQ-short; Watson et al., 1995), the Perceived Stress Scale (PSS; Cohen et al., 1983), the Temporal Experience of Pleasure Scale (TEPS; Gard et al., 2006), and the Behavioral Inhibition and Behavioral Activation Scales (BIS/BAS; Carver and White, 1994).

Twenty minutes after arrival, the first of three saliva samples was collected to measure baseline cortisol levels. Next, participants completed the first set of “in-the-moment” state self-report questionnaires to obtain baseline ratings of their current mood (=“baseline” timepoint for analyses). These included the state versions of the State Trait Anxiety Inventory (STAI-S; Spielberger et al., 1983) and the Positive and Negative Affect Schedule (PANAS-S; Watson et al., 1988).

Next, the second written informed consent was obtained using either a “no stress” condition or a “stress” condition consent form. The “stress” consent form stated that participants might receive electrical shocks (via two electrodes attached to their right hand) during two ensuing computer games: “up to two” shocks during the first task (a “filler” task) and “up to three” shocks during the second task (the PSST). Participants then completed a computerized basic attention task that acted as a “filler” task, during which all participants in the “stress” condition received one electrical shock (performance in this task was extraneous to study hypotheses). This task served the purpose of making the potential for shock a credible threat given that we did not actually administer any shock during the main task of interest (PSST). Following the “filler” task, participants completed a second identical set of “in-the-moment” state self-report questionnaires (=“post-filler-task/pre-PSST” timepoint); additionally, participants were asked to provide a second saliva sample for cortisol level analyses (approximately 13 min after the shock).

Thereafter, participants who completed the “filler” task in the “stress” condition were further subdivided into “controllable stress” and “uncontrollable stress” conditions, and participants received the appropriate set of instructions for the PSST. Between the training and test phases of the PSST, participants completed a third set of “in-the-moment” state self-report questionnaires (=“PSST” timepoint) probing affect experienced during the training phase of the task (i.e., the phase of the task involving the stress manipulation). Following the test phase of the PSST, participants were asked to provide a third saliva sample for cortisol analyses (time-locked to 10 min from the end of the training phase of the PSST in order to capture cortisol levels when participants in the stress conditions were under perceived “threat of shock”). Then, they completed a final set of “in-the-moment” state self-report questionnaires (=“post-task” timepoint). Participants also completed a post-task questionnaire to probe their experiences during the session. At the end of the experiment, all participants were debriefed and either paid ($10/h) or awarded study credit for their time. The overall session took approximately 1.5–2 h, and subjects received $15–20 or 1.5–2 study credits. Please see Appendix for detailed descriptions of trait and state measures.

#### Stress manipulation

Two electrodes were placed on the right hand of each participant assigned to either of the stress conditions, and the electrode wires were attached to a shock box placed on the table in front of the participant. The shock level was adjusted to what each participant felt was “aversive, but not painful.” This was done by beginning at the lowest level of shock intensity and having the participant experience a brief shock at each level to have the participant identify a level that she felt was “aversive, but not painful.” The maximum current intensity (4 mA; Coulbourn E13–22) was approved by the local IRB. Prior to the “filler” task, these participants were told that they could receive up to two electrical shocks, but the task was actually programmed to administer only one shock. In the PSST, all participants were told they would see a multicolored bar on either side of the computer screen with a tick mark that would periodically move up and down. In the “no stress” condition, they were told that the bars had no meaning. They were also told that occasionally the border of the computer screen would flash red and they should press down on a foot pedal when they saw this visual cue in order to indicate that they were attending to the task. The task was programmed for the cue to appear 1–2 times during each practice block, but participants were not given information about the frequency of this occurrence. For participants in both the “controllable stress” and “uncontrollable stress” conditions, the border flashing red indicated that a shock might occur in the next 15–30 s and they were told that the location of the tick mark within the multicolored bars would indicate the likelihood they would receive a shock. For these participants, the multicolored bars were labeled with “danger” at the top and “safe” at the bottom, and the closer the tick mark was to the top of the bar, the higher the likelihood of receiving a shock. Moreover, participants in the stress conditions were told that the movement of the tick mark was determined by the computer and was unrelated to their performance on the task. However, participants in the “controllable stress” condition were told that pressing the foot pedal when they saw the red border visual cue would override the computer and lower the location of the tick mark in the bars, thus reducing (albeit not fully eliminating) the likelihood they would receive a shock. When these participants pressed down on the foot pedal, the tick mark did shift down closer to the “safe” zone at the bottom of the bar, providing some visual feedback. In contrast, participants in the “uncontrollable stress” condition were instructed to press down on the foot pedal to indicate they were attending to the task (i.e., they received the same instructions about the foot pedal as those in the “no stress” condition) and this had no effect on the location of the tick mark. Participants in both stress conditions were told they could receive up to three electrical shocks during the PSST; in reality, no shock was administered during this task. Of note, the threat-of-shock stress manipulation was only in effect during the training phase of the PSST. This was the target of our stress manipulation because reward and punishment feedback were only provided during that phase of the task.

#### “Filler” task

Participants completed a brief version (~8 min) of a Continuous Performance Task (CPT; Conners, 1995) as a “filler” task. They were presented with a series of letters (“O,” “T,” “H,” “Z,” or “X”) on a computer screen, one at a time, and were instructed to press the space bar immediately following any letter except for “X.” Participants completed two blocks of 125 trials, with each letter appearing in 25 trials; on each trial, the letter stimulus was presented for 500 ms, followed by an interstimulus interval that varied between 1250–1550 ms.

#### Modified probabilistic stimulus selection task (PSST)

**Figure 2:**
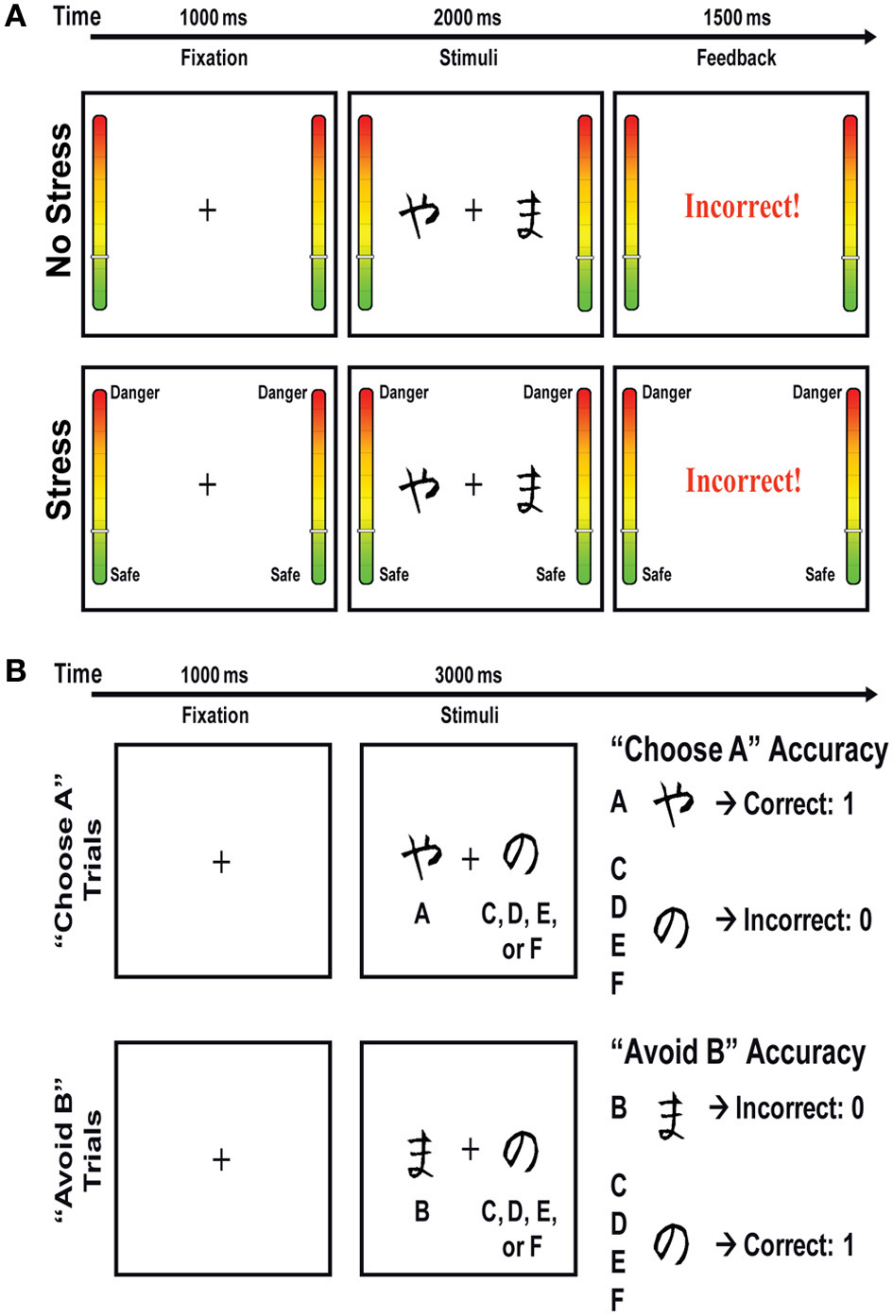
**(A)** Schematic representation of the *training* phase of the Probabilistic Stimulus Selection Task, which was performed under stress or no stress conditions. In the no-stress condition, every time a red border flashed, participants were instructed to press a foot pedal to indicate they were attending to the task. In the two stress conditions, participants were told that the border flashing red indicated a shock might occur in the ensuing 15–30 s. In the controllable stress condition, participants were further instructed that they could reduce (though not fully eliminate) the likelihood of the shock if they pressed the foot pedal when they saw the red border cue. In contrast, participants in the “uncontrollable stress” condition were instructed to press the foot pedal to indicate they were attending to the task. **(B)** Schematic representation of the *test* phase of the Probabilistic Stimulus Selection Task. No stress was presented during this phase.

The PSST included a training phase and a test phase (Figure 2). During the training phase, participants were presented with three different stimuli pairs (AB, CD, EF) in random order, and were instructed to choose one of the two stimuli by pressing one of two response buttons. Following a subject's response, feedback was given to indicate whether the choice was “correct” or “incorrect.” Importantly, this feedback was probabilistic, such that for AB trials, a choice of stimulus A led to correct (positive) feedback in 80% of the trials, while a choice of stimulus B led to incorrect (negative) feedback in these trials (with the relations reversed for the other 20% of AB trials). The stimulus pair CD was less reliable, with stimulus C correct in 70% of CD trials, and the stimulus pair EF was the least reliable, with stimulus E correct in 60% of the EF trials. During this training phase, subjects learned to choose stimuli A, C, and E more frequently than B, D, or F. Of note, selection of A over B could be achieved either by learning that choosing A usually leads to positive feedback or learning that choosing B usually leads to negative feedback, or both. Participants completed the training phase either under a “no stress,” “controllable stress,” or “uncontrollable stress” condition. The training phase was terminated after participants reached performance criteria (65% A in AB, 60% C in CD, and 50% E in EF) or after the completion of six blocks. The performance criteria were set so that all participants would be at approximately the same performance level before proceeding to the test phase (i.e., there was no “overtraining” for subjects who had already learned the contingencies because they would advance to the test phase earlier).

In the test phase, subjects were presented with the same three stimuli pairs, as well as all novel combinations of stimuli pairs, and feedback was not provided (Figure 2). In order to examine whether subjects learned more about the positive or negative outcomes of their decisions in the training phase, the stimuli pairs of primary interest in the test phase were those involving an A or B stimulus paired with a novel stimulus (e.g., AC, AD, AE, and AF; BC, BD, BE, and BF), referred to as “transfer pairs.” These transfer pairs enabled assessment of the degree to which participants learned from prior positive feedback to choose the most reinforced stimulus (“Choose A”) and/or learned from prior negative feedback to avoid the most punished stimulus (“Avoid B”). Prior studies have shown that these conditions are differentially sensitive to dopaminergic manipulation and that performance in the “Choose A” condition is correlated with neural responses to positive outcomes, whereas performance in the “Avoid B” condition is correlated with neural responses to negative outcomes.

The stimuli presented in the PSST were black-and-white Hiragana characters. In the training phase, each trial began with a fixation cross in the middle of the screen for 1000 ms, followed by a stimuli pair for 2000 ms or until the participant made a response. Thereafter, visual feedback was provided for 1500 ms as either “Correct” in blue letters, “Incorrect” in red letters, or “No response detected” in red letters (if the subject did not respond within 2000 ms). Each block of the training phase had 60 trials with 20 trials per stimuli pair. In the test phase, each trial began with a fixation cross for 1000 ms, followed by a stimuli pair for 3000 ms or until the participant made a response. The test phase consisted of one block of 90 trials, with six trials of each of the 15 possible stimuli pairs.

#### Saliva samples

For saliva collection, participants were instructed to put a small cotton roll (Salivette) in their mouth for approximately 90 s, and then place the saliva-soaked cotton into a small plastic tube. Saliva samples were subsequently stored in a freezer (≤ −20 degrees Celsius) until assayed. The timing of the collection of cortisol samples (specified in the Procedures section above) was based on prior research indicating that cortisol typically peaks about 10–20 min after stressor onset (e.g., Kudielka et al., 2004). To control for diurnal rhythms in cortisol levels, all participants were run between the hours of 1 and 6 pm (Dickerson and Kemeny, 2004). To further control for fluctuations in hormone levels, participants were asked to adhere to the following instructions: no eating or brushing their teeth for at least an hour before the session; no consumption of yogurt for at least 2 h before the session; no consumption of any caffeine-containing products or alcohol the day of the session; no strenuous exercise the day of the session. Information was also collected regarding the time of day participants woke up and the time of the session.

### Data analyses

#### Trait and dispositional self-report measures

Total and subscale scores were computed for the BDI, MASQ, PSS, TEPS, and BIS/BAS, and *t*-tests were run to compare participants who completed the task under “stress” vs. “no-stress” conditions.

#### “In-the-moment” state self-report measures

To assess the effectiveness of the stress manipulation, separate mixed ANOVAs were conducted on STAI-S, PANAS-PA (positive affect), and PANAS-NA (negative affect) scores, with *Time* (Baseline, PSST) as a repeated measure and *Group* (Stress, No-Stress) as a between-subjects factor. Significant findings were followed up with *t*-tests.

#### PSST training phase

To evaluate potential group differences in training, *t*-tests were conducted to compare groups on the number of blocks required to reach performance criteria; separate mixed ANOVAs were run for accuracy and RT on the final training block with *Trial Type* (AB, CD, EF) and *Group* as factors. Significant differences were followed up with *t*-tests.

#### PSST test phase

Prior to the main analyses of interest, a *t*-test was run to compare accuracy on AB trials (the “easiest” trial type) in the test phase to confirm that there were no significant differences between “stress” and “no stress” groups with regard to participants learning the basic task. Although the performance criteria in the training phase was intended to address this issue, it is possible that participants could have become confused by the lack of feedback and the addition of novel stimuli pairs in the test phase, so this served to verify that learning carried over to the test phase.

Thereafter, to assess whether participants learned more from the positive or negative feedback they received during training, data from the test phase were analyzed with respect to performance on the test trials involving novel combinations of stimuli pairs that included either an A or a B stimulus, respectively. For trials involving an A stimulus paired with a novel stimulus (“Choose A” trials), accuracy was calculated as the proportion of trials on which the participant chose A (the most frequently reinforced stimulus) over the novel stimulus. For trials involving a B stimulus paired with a novel stimulus (“Avoid B” trials), accuracy was calculated as the proportion of trials on which the participant avoided B (the most frequently punished stimulus) and chose the novel stimulus instead. Next, ANOVAs were performed with *Trial Type* (“Choose A,” “Avoid B”) and *Group* as factors to examine accuracy and RT separately. Significant differences were followed up with the appropriate *t*-tests.

#### Saliva samples (cortisol)

In order to obtain cortisol levels, saliva samples were sent to the Laboratory for Biological Health Psychology (Brandeis University, MA, USA) and analyzed in a single batch to avoid assay variability (intra-assay CV = 6.48%; inter-assay CV = 6.06%). These values were then entered into an ANOVA using *Time* (T1 = baseline, T2 = post-“filler”-task/pre-PSST, T3 = post-PSST) and *Group* as factors. Given the diurnal drop in cortisol levels throughout the day (Schmidt-Reinwald et al., 1999), and the inevitable variability in wake-up time across participants, we also calculated the difference between waking time and time of the first saliva collection; this value was used as a covariate in the aforementioned ANOVA. Next, in line with previous studies (e.g., Townsend et al., 2011), we calculated cortisol reactivity scores (i.e., difference scores from T1 to T2, or T1 to T3) for all participants. Finally, an ANOVA was run to compare cortisol reactivity scores with *Group*.

#### Follow-up analyses: using changes in cortisol levels and self-reported state anxiety to identify a stress-reactive subgroup

Given that “threat of shock” might only have been stressful for a sub-group of participants, we identified individuals who were relatively high stress responders based on changes in cortisol levels and self-reported state anxiety from T1 (baseline) to T2 (~13 min after subjects received the shock administered in the “filler” task). Initially, we examined descriptive statistics on the distribution of cortisol reactivity scores from T2-T1 within “no-stress” and “stress” groups to examine if there was indeed considerable variability in reactivity scores within each group. In order to obtain a new “stress reactive” group with only stress-reactive participants, we first standardized the T2-T1 cortisol reactivity scores across all participants. Next, using these standardized values, participants were divided into three tiers: high responders (>0.24), medium responders (−0.27 ≥ and ≤ 0.24), and low responders (< −0.27). These cut-off scores were selected so that approximately 1/3 of participants were in each tier. Similarly, we standardized the T2-T1 change scores in self-reported state anxiety levels (using STAI scores), and again divided participants into three tiers: high responders (>0.44), medium responders (−0.66 ≥ and ≤ 0.44), and low responders (< −0.66). Thereafter, a new “stress reactive” group was created that included only participants who completed the task under stress *and* were relatively high stress responders, defined as being in the “high responder” tier with regard to both changes in cortisol levels and self-reported state anxiety. Using this new “stress reactive” group, all of the aforementioned analyses were re-run to compare the “stress reactive” and “no-stress” groups on demographics, trait and state self-report measures, and performance on the PSST task.

## Results

### Trait and dispositional self-report measures (no-stress vs. stress groups)

As evident in Table 1, there were no significant differences between the “no-stress” and “stress” groups on the trait or dispositional self-report measures collected at baseline (all *t*s ≤ 1.67, *p*s ≥ 0.10). Accordingly, putative differences in behavioral performance or stress reactivity were not confounded by group differences in trait or dispositional affect, or ongoing stress levels.

**Table 1:** **Demographics, trait and dispositional self-report measures by groups**.

	**No stress (NS) group (*n* = **27**)**	**Stress (S) group (*n* = **68**)**	**Stress reactive (SR) group (*n* = **18**)**	**NS vs. S statistic**	***p***	**NS vs. SR statistic**	***P***
Gender (% female)	100%	100%	100%	N/A	N/A	N/A	N/A
Age (years)	21.43 (±1.79)	21.32 (±2.20)	22.05 (±1.92)	*t*_(93)_ = 0.22	0.83	*t*_(43)_ = 1.11	0.28
Education (years)	14.81 (±1.39)	14.35 (±1.61)	14.94 (±1.35)	*t*_(93)_ = 1.31	0.19	*t*_(43)_ = 0.31	0.76
Marital status (% single)	100%	93%	89%	χ^2^(2) = 2.10	0.35	χ^2^(1) = 3.14	0.08
Income^*^ (% < $50,000)	90%	74%	69%	χ^2^(1) = 2.29	0.13	χ^2^(1) = 2.29	0.13
Compensation form (% monetary)	85%	90%	78%	χ^2^(1) = 0.39	0.54	χ ^2^(1) = 0.41	0.52
Ethnicity (% Caucasian)	85%	59%	61%	χ^2^(2) = 10.07	**0.01**	χ^2^(1) = 3.39	0.07
BDI-II	1.85 (±2.38)	2.21 (±2.34)	1.67 (±2.03)	*t*_(93)_ = −0.66	0.51	*t*_(43)_ = 0.27	0.79
MASQ: GDA	15.52 (±4.74)	15.66 (±3.90)	16.22 (±3.21)	*t*_(93)_ = −0.15	0.88	*t*_(43)_ = −0.55	0.59
MASQ: GDD	16.85 (±5.25)	18.10 (±5.12)	17.72 (±5.79)	*t*_(93)_ = −1.07	0.29	*t*_(43)_ = −0.52	0.60
MASQ: AA	20.52 (±4.82)	19.59 (±3.62)	19.28 (±3.05)	*t*_(93)_ = 1.03	0.31	*t*_(43)_ = 0.97	0.34
MASQ: AD	49.56 (±10.90)	49.71 (±10.68)	45.83 (±8.99)	*t*_(93)_ = −0.06	0.95	*t*_(43)_ = 1.20	0.24
Perceived stress scale	19.67 (±6.33)	20.68 (±5.86)	20.83 (±4.62)	*t*_(93)_ = −0.74	0.46	*t*_(43)_ = −0.67	0.51
TEPS: anticipatory	64.67 (±6.68)	64.65 (±9.78)	66.11 (±7.80)	*t*_(93)_ = 0.01	0.99	*t*_(43)_ = −0.67	0.51
TEPS: consummatory	48.41 (±5.56)	50.66 (±6.06)	52.22 (±5.70)	*t*_(93)_ = −1.67	0.10	*t*_(43)_ = −2.23	**0.03**
BIS/BAS: reward responsiveness	7.48 (±1.67)	7.51 (±2.18)	7.56 (±2.09)	*t*_(93)_ = −0.07	0.94	*t*_(43)_ = −0.13	0.90
BIS/BAS: drive	9.19 (±1.96)	9.06 (±2.13)	9.06 (±1.73)	*t*_(93)_ = 0.27	0.79	*t*_(43)_ = 0.23	0.82
BIS/BAS: fun seeking	8.04 (±2.16)	7.78 (±2.23)	8.00 (±2.47)	*t*_(93)_ = 0.51	0.61	*t*_(43)_ = 0.05	0.96
BIS/BAS: inhibition	16.00 (±2.82)	15.40 (±2.83)	15.33 (±2.74)	*t*_(93)_ = 0.94	0.35	*t*_(43)_ = 0.79	0.44

BDI-II, Beck Depression Inventory-II; MASQ, Mood and Anxiety Symptom Questionnaire; GDA, General Distress Anxious; GDD, General Distress Depressive; AA, Anxious Arousal; AD, Anhedonic Depression; TEPS, Temporal Experience of Pleasure Scale; BIS/BAS, Behavioral Inhibition and Behavioral Activation Scales.

* *Participants who chose not to report income are not included in the Income statistics; 7 out of 27 (26%) “no stress” participants and 15 out of 68 (22%) “stress” participants chose not to report income. The bold values serve to highlight statistically significant values*.

### “In-the-moment” state self-report measures (no-stress vs. stress groups)

Analyses of both state anxiety (STAI-S scores) and negative affect (PANAS-NA scores) revealed similar effects: significant *Time × Group* interactions [*F*s_(1, 93)_> 5.06, *p*s < 0.03], along with significant main effects of *Time* [*F*s_(1, 93)_ > 8.80, *p*s < 0.01] and *Group* [*F*s_(1, 93)_> 4.87, *p*s ≤ 0.03]. Importantly, at baseline, groups did not differ in their levels of state anxiety or negative affect [*t*s_(93)_< 0.46, *p*s > 0.64]. During the PSST, participants in the “stress” group reported significantly higher levels of state anxiety and negative affect than participants in the “no-stress” group [*t*s_(93)_> 3.00, *p* < 0.01]. Within-group paired *t*-tests indicated that anxiety increased from baseline to PSST in the “no stress” group [*t*_(26)_ = 2.17, *p* = 0.04] and, to a much greater degree, in the “stress” group [*t*_(67)_ = 8.54, *p* < 0.01]. Meanwhile, negative affect increased significantly from baseline to PSST in the “stress” group [*t*_(67)_ = 4.45, *p* < 0.01] but not in the “no stress” group [*t*_(26)_ = 0.62, *p* = 0.54]. The mixed ANOVA on PANAS-PA scores revealed only a significant main effect of *Time* [*F*_(1, 93)_ = 11.33, *p* < 0.01; all other *F*s < 2.58, *p*s > 0.11], with levels of positive affect decreasing from baseline to PSST in both groups.

### PSST training phase (no-stress vs. stress groups)

Groups did not differ in the number of completed training blocks [*t*_(93)_ = 0.27, *p* = 0.79]; all groups took approximately three blocks to advance to the test phase (No-Stress: 3.15 ± 1.75; Stress: 3.25 ± 1.62). A *Trial Type* (AB, CD, EF) × *Group* (“no stress,” “stress”) mixed ANOVA on accuracy scores in the final training block indicated only a significant main effect of *Trial Type* [*F*_(1, 93)_= 24.71, *p* < 0.01; all other *F*s < 2.41, *p*s > 0.12]; as expected, participants were most accurate on the AB trial type and least accurate on the EF trial type. No significant differences emerged from the mixed ANOVA for RT in the final training block (all *F*s < 1.06, *p*s > 0.30). Altogether, these findings indicate that (1) the probabilistic contingencies elicited the intended behavioral effects, and (2) groups did not differ in performance during the training phase.

### PSST test phase (no-stress vs. stress groups)

The groups did not differ significantly in their accuracy on AB trials in the test phase [No-Stress Group = 90% (±12%); Stress Group = 86% (±23%); [*t*_(93)_= 0.94, *p* = 0.35]], confirming that learning carried over to the test phase similarly for the two groups. Contrary to hypotheses, the *Trial Type* (“Choose A,” “Avoid B”) × *Group* ANOVA on accuracy scores revealed no significant effects (all *F*s < 1.82, *p*s > 0.17).

For RT scores, the analogous *Trial Type* × *Group* ANOVA yielded a significant main effect of *Trial Type* [*F*_(1, 93)_ = 29.52, *p* < 0.01] and a trend for a *Trial Type* × *Group* interaction [*F*_(1, 93)_ = 3.29, *p* = 0.07]. These results reflected both groups being faster on “Choose A” trials than “Avoid B” trials, with the “no-stress” group demonstrating this pattern to a greater extent.

### Stress-reactive subgroup (defined by changes in cortisol levels and self-reported state anxiety)

An examination of descriptive statistics on the distribution of cortisol reactivity scores at T2-T1 within “no-stress” and “stress” groups revealed considerable variability in reactivity scores within each group: scores in the “no stress” group ranged from −5.51 to 1.71 (mean: −1.56 ± 1.57); scores in the “stress” group ranged from −7.82 to 11.78 (mean: −0.95 ± 2.40). Per design, cortisol reactivity scores at T2-T1 were significantly higher in the new “stress reactive” group than the “no-stress” group [*t*_(42)_ = 4.01, *p* < 0.01; degrees of freedom reduced by 1 because cortisol data was missing for one subject at T2]. Importantly, cortisol reactivity scores at T3-T1 continued to be significantly higher in the “stress reactive” group than the “no-stress” group [*t*_(41)_ = 3.75, *p* < 0.01; degrees of freedom reduced by 2 because cortisol data missing for two subjects at T3], suggesting that subjects in the “stress reactive” group continued to be more physiologically stressed during the PSST than subjects in the “no stress” group. The new groups did not differ significantly from each other on any of the following demographic variables: gender, age, years of education, marital status, income level, form of compensation, or ethnicity (see Table 1).

### Trait and dispositional self-report measures (no-stress vs. stress-reactive groups)

As compared to the “no-stress” group, the “stress reactive” group reported significantly higher scores on the consummatory subscale of the Temporal Experiences of Pleasure Scale (TEPS), which assesses individual trait dispositions in consummatory experiences of pleasure [*t*_(43)_ = 2.23, *p* = 0.03; all other *t*s_(43)_ ≤ 1.36, *p*s ≥ 0.18]. Due to this finding, the TEPS consummatory subscore was used as a covariate.

### “In-the-moment” state self-report measures (no-stress vs. stress-reactive groups)

#### State anxiety

**Figure 3:**
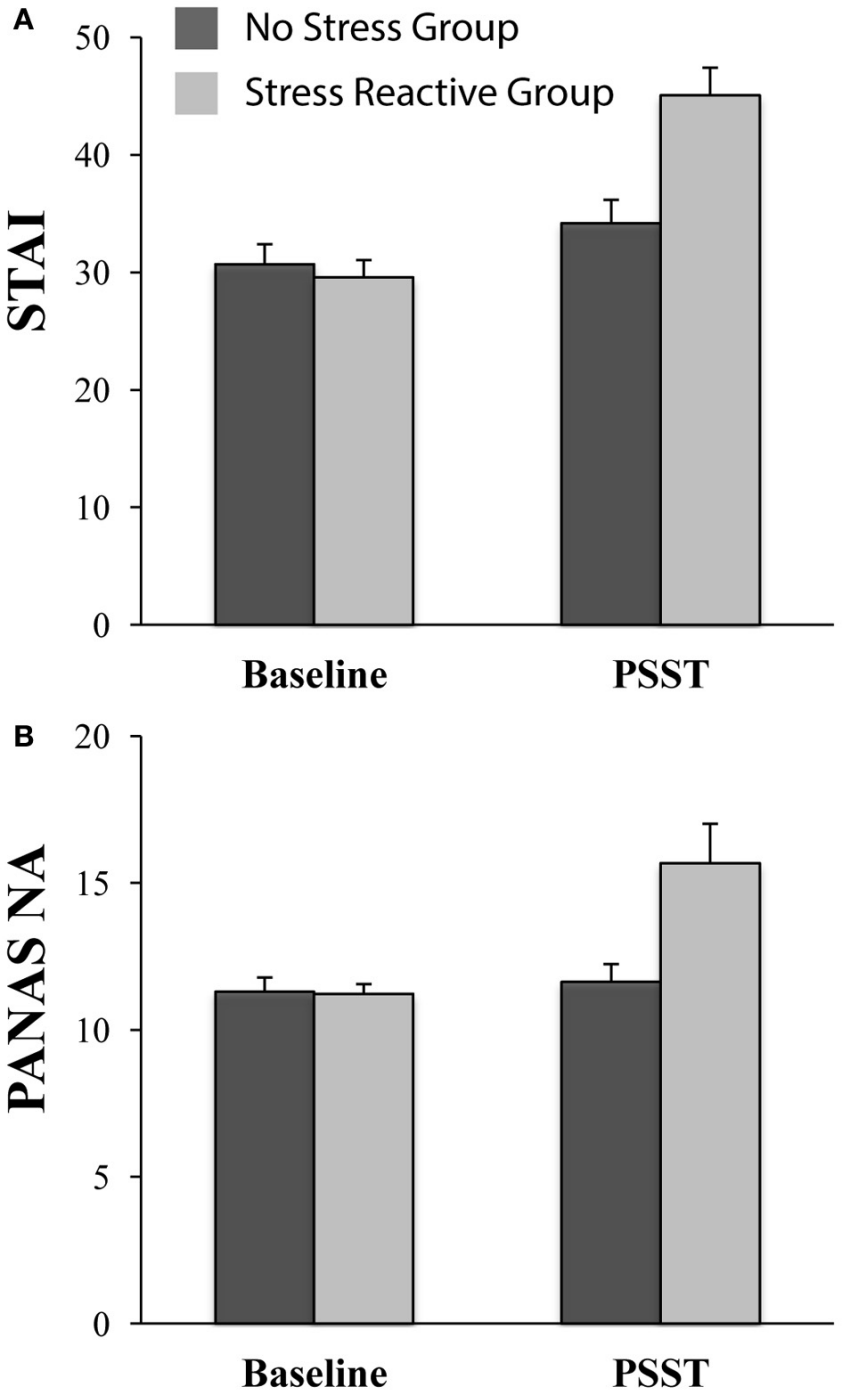
**Affective ratings in the no-stress (*n* = 27) and stress-reactive (*n* = 18) groups at baseline and during the PSST. (A)** State Trait Anxiety Inventory (STAI) scores; and **(B)** Negative Affect score on the Positive and Negative Affect Schedule (PANAS). For both scales, the state version was used.

As shown in Figure 3, and in line with the new group design, the ANCOVA on STAI-S scores revealed only a significant *Time × Group* interaction [*F*_(1, 42)_ = 13.33, *p* < 0.01], whereas the *Time* [*F*_(1, 42)_ = 0.29, *p* = 0.59] and *Group* [*F*_(1, 42)_ = 3.52, *p* = 0.07] effects were not significant. At baseline, groups did not differ in their state anxiety levels [*t*_(43)_ = −0.48, *p* = 0.63]. During the PSST, participants in the “stress reactive” group reported significantly higher levels of state anxiety than participants in the “no-stress” group [*t*_(43)_ = 3.57, *p* < 0.01]. Within-group paired t-tests indicated that anxiety increased from baseline to PSST in both the “stress reactive” group [*t*_(17)_ = 6.31, *p* < 0.01] and “no stress” group [*t*_(26)_ = 2.17, *p* = 0.04].

#### State negative affect

The ANCOVA on PANAS-NA scores indicated only a significant *Time × Group* interaction [*F*_(1, 42)_ = 6.00, *p* = 0.02]; *Time* [*F*_(1, 42)_ = 0.95, *p* = 0.33] and *Group* [*F*_(1, 42)_ = 3.57, *p* = 0.07]; see Figure 3. At baseline, groups did not differ in their levels of negative affect [*t*_(43)_ = −0.12, *p* = 0.90]; during the PSST, the “stress reactive” group reported significantly more negative affect than the “no stress” group [*t*_(43)_ = 2.90, *p* < 0.01]. Paired *t*-tests indicated that negative affect increased significantly from baseline to PSST in the “stress reactive” group [*t*_(17)_ = 3.03, *p* < 0.01], but not in the “no stress” group [*t*_(26)_ = 0.62, *p* = 0.54].

#### State positive affect

The ANCOVA revealed no significant effects (all *F*s < 1.95, *p*s > 0.17).

### PSST training phase (no-stress vs. stress-reactive groups)

Groups did not differ in the number of completed training blocks [*t*_(43)_ = 0.57, *p* = 0.58]; all groups took approximately three blocks to advance to the test phase (No-Stress: 3.15 ± 1.75; Stress-Reactive: 3.44 ± 1.69). Separate *Trial Type* (AB, CD, EF) × *Group* (“no stress,” “stress reactive”) ANCOVAs on accuracy scores and RT scores revealed no significant effects (all *F*s < 3.13, all *p*s > 0.08).

### PSST test phase (no-stress vs. stress-reactive groups)

**Figure 4:**
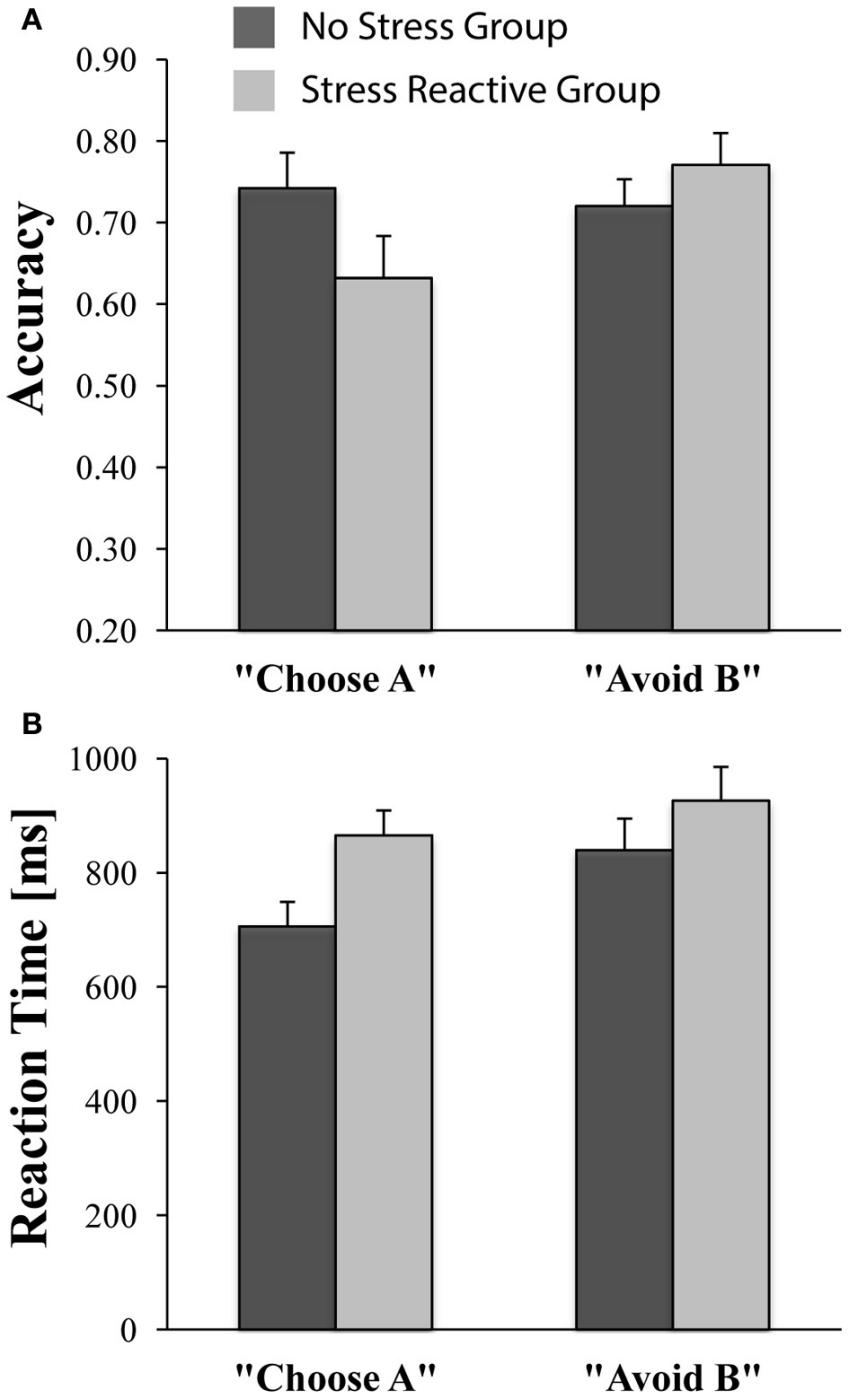
**Performance on “Choose A” and “Avoid B” trials during the PSST test phase in the no-stress (*n* = 27) and stress-reactive (*n* = 18) groups. (A)** Accuracy; **(B)** Reaction Time (in ms).

The ANCOVA comparing accuracy on AB trials in the test phase with *Group* (“no stress,” “stress reactive”) revealed no significant group differences [No-Stress Group = 90% (±12%); Stress-Reactive Group = 92% (±16%); [*F*_(1, 42)_ = 0.63, *p* = 0.43], confirming that learning carried over to the test phase similarly for the two groups. Critically, the *Trial Type* (“Choose A,” “Avoid B”) × *Group* (“no stress,” “stress reactive”) ANCOVA on accuracy scores revealed a main effect of *Trial Type* [*F*_(1, 42)_ = 5.72, *p* = 0.02], which was qualified by a significant *Group* × *Trial Type* interaction [*F*_(1, 42)_ = 6.45, *p*= 0.015], whereas the *Group* main effect was not significant [*F*_(1, 42)_ = 0.14, *p* = 0.71]. As shown in Figure 4, these findings indicate that the “stress reactive” group displayed relatively lower accuracy on reward-related trials than punishment-related trials compared to the “no stress” group, which exhibited the opposite pattern.

For RT, an analogous *Group* × *Trial Type* ANCOVA yielded only a significant main effect of *Group* [*F*_(1, 42)_ = 7.59, *p* < 0.01; all other *p*s > 0.18], due to faster RTs in the “no-stress” group than the “stress reactive” group (Figure 4). Follow-up analyses indicated that, compared to the “no stress” group, participants in the “stress reactive” group demonstrated significantly slower RTs on the “Choose A” trials [*F*_(1, 42)_ = 13.67, *p* < 0.01], but not the “Avoid B” trials [*F*_(1, 42)_ = 3.13, *p* = 0.08]. Moreover, participants within the “no stress” group were faster on their “Choose A” trials than their “Avoid B” trials [*t*_(26)_ = −4.47, *p* < 0.01], suggestive of a reward-related RT bias, whereas those in the “stress reactive” group had similar RTs on both trial types [*t*_(17)_ = −1.41, *p* = 0.18] and did not show this effect.

## Discussion

This study was designed to extend our understanding of stress-related anhedonic behavior by examining whether stress specifically reduces reward processing (i.e., learning from positive feedback) or more generally influences incentive processing (i.e., learning from both positive and negative feedback). The stress manipulation induced significantly higher levels of negative affect and anxiety in those individuals who completed the PSST under stress vs. no-stress conditions. Yet, contrary to our hypotheses, the stress manipulation did not have a significant differential impact on cortisol reactivity or task performance at the group level, likely due to large individual differences. Importantly, however, individuals with heightened cortisol reactivity and increased negative affect following acute stress did demonstrate deficits specific to reward processing. These latter findings suggest that, in highly stress-reactive individuals, stress may selectively result in reward processing deficits with no reduction in punishment processing.

Given that the “threat-of-shock” stressor did evoke significantly higher levels of self-reported negative affect and anxiety in the “stress” group than the “no-stress” group, which was in line with prior independent studies (Bogdan and Pizzagalli, 2006; Bogdan et al., 2011), we were surprised to find that the “stress” group did not demonstrate significantly higher levels of cortisol reactivity. In light of these patterns, it is possible that our stress manipulation may not have elicited as strong of a physiological stress response as intended because only a single shock was administered during the “filler” task and none were administered during the PSST. In addition, the stress manipulation did not include any social evaluative component, which has been shown to reliably produce physiological stress responses (Kirschbaum et al., 1993). Moreover, for participants in the “stress” group, the border of the computer screen flashing red during the PSST indicated that a shock could occur in the next 15–30 seconds; it is possible that this cue may have reduced the stressfulness of the “threat-of-shock” by increasing the perceived predictability of the stressor. In fact, predictable stressors typically elicit smaller physiological stress responses and are experienced as less aversive than unpredictable stressors (Anisman and Matheson, 2005). In light of these null cortisol findings, it was not entirely surprising that initial analyses of task performance across groups yielded no significant between-group differences during the training or test phases of the PSST.

One potential explanation for the lack of significant findings in this initial set of analyses may be that there was a broad range of individual differences within the group of individuals who completed the task under stress in terms of how physiologically “stressed out” participants became in response to the “threat-of-shock.” An examination of cortisol reactivity scores within each group indeed confirmed that there was substantial intra-group variability. Accordingly, we conducted follow-up analyses by identifying a stress-reactive subgroup based on cortisol reactivity as well as self-reported anxiety levels; the new “stress reactive” group included only those participants who completed the task under stress and were “high responders” from both a physiological (cortisol levels) and self-reported experiential (STAI scores) perspective. In line with these demarcations, the new “stress reactive” group also demonstrated a significant increase in negative affect (PANAS-NA scores) that was not apparent in the “no stress” group, reinforcing coalescence between biological measures and self-report measures of stress response.

### Stress-sensitive individuals demonstrate reward-specific impairments

Consistent with previous studies (Bogdan and Pizzagalli, 2006; Pizzagalli et al., 2007; Bogdan et al., 2010), and our main hypotheses, participants in the new “stress reactive” group demonstrated reduced reward sensitivity relative to participants in the “no-stress” group. This was supported in the following ways: first, there was a significant *Group* (“no stress,” “stress reactive”) × *Trial Type* (“Choose A,” “Avoid B”) interaction for accuracy during the test phase of the PSST, which was due to relatively lower accuracy on reward-related (“Choose A”) trials than punishment-related (“Avoid B”) trials in the “stress reactive” group, compared with the opposite pattern exhibited by the “no-stress” group (i.e., relatively higher accuracy on reward-related than punishment-related trials). This finding suggests that stress-sensitive participants did not experience a global decrease in accuracy on the task under stress, but rather a more specific reduction in accuracy on reward-related trials only. This reward-processing deficit may reflect reduced sensitivity to positive feedback (during the training phase of the PSST), evident in an impaired ability to use this reward information to guide decision making in novel contexts (during the test phase of the PSST). Secondly, participants in the “no-stress” group demonstrated a reward-related RT bias that was absent in the “stress reactive” group. Specifically, the “no stress” group demonstrated faster RTs on reward-related trials than punishment-related trials, while the RTs of the “stress reactive” group were not significantly different between trial types. Moreover, participants in the “no-stress” group were significantly faster than participants in the “stress reactive” group on the reward-related trials but not the punishment-related trials. Importantly, these findings suggest that speed-accuracy tradeoffs did not play a significant role in the present results. For example, the fact that the “stress reactive” group, as compared to the “no stress” group, had poorer accuracy *and* slower RTs on reward-related trials runs counter to the notion that poorer accuracy could have been due to a speed-accuracy tradeoff of faster RTs. Overall, our results expand prior lines of research on stress-induced reductions in reward responsiveness by suggesting that stress may selectively reduce sensitivity to reward feedback and does not more broadly reduce sensitivity to feedback in general.

During the test phase, there were no group differences in accuracy on the most salient trials from the training phase (e.g., AB trials), which (1) suggests that all participants learned the basic task and this learning carried over to the test phase, and (2) provides further evidence that stress did not induce a global performance deficit across the task (e.g., differences only emerged for novel trial types in the test phase). These findings, in combination with the fact that participants across groups needed a comparable number of training blocks to reach performance criteria during the training phase, also suggest that results were not likely the byproduct of psychometric artifacts. More specifically, as highlighted in experiments assessing the effects of threat on working memory performance (Shackman et al., 2006), it is important to address whether results could be merely the artifact of an additional load on attentional resources in the stress condition, rather than stress *per se*. If this were the case, however, we would expect to see global deficits in task performance for individuals who completed the task under stress. In addition, a predominant lack of group differences on trait and dispositional self-report measures (the one exception being the consummatory subscale of the TEPS, which was controlled for in the analyses), and no group differences at baseline on any affective state self-report measures, suggests that putative differences in behavioral performance or stress reactivity were not confounded by group differences in affect, mood, or ongoing life stress.

In related research that warrants acknowledgement, Lighthall and colleagues (2013) recently reported that participants who completed a PSST *after* exposure to a cold pressor stress manipulation had relatively reduced punishment learning and increased reward learning. However, the stressor was terminated well before the beginning of the PSST (and an unrelated memory task was administered between the stressor and the PSST); this sequence of events raises the possibility that their observed results may have stemmed from “relief” experienced by participants after the stressor. In line with the conceptualization of “stress relief” as rewarding, “relief” from stressors has been recently associated with activation of reward-related neural regions (Leknes et al., 2011) and increased dopamine levels (Navratilova et al., 2012). Clearly, more research is needed to examine the putative relationship between negative stressors and decreased reward sensitivity, with particular focus on the temporal unfolding of such processes.

### Limitations

There are several limitations to the current study that should be acknowledged. First, the study included only female participants due to sex differences in psychological and hormonal responses to stress (e.g., women demonstrate a more pronounced stress response than men; Nolen-Hoeksema and Hilt, 2009). Thus, future studies will be required to determine if the current stress-induced reward-specific deficits generalize to males. Second, the strength of findings is limited by the fact that significant between-group results only emerged after re-running the main analyses of interest using a “stress reactive” subgroup defined based on physiological and self-reported experiential indices of stress responsiveness. This new “stress reactive” group had a relatively small sample size and contained participants who had received two different sets of instructions regarding controllability of the stressor. However, the lack of significant differences between these participants (with regard to both self-report and physiological measures; see Appendix Analyses) mitigates the potential effect of this latter limitation. Third, it is important to acknowledge the inherently limited ecological validity of an acute “threat-of-shock” laboratory stressor and the potentially diminished strength of laboratory stressors that do not include a social evaluative component. Fourth, given that findings from this study pertain to learning from positive vs. negative feedback, it remains to be seen whether the patterns found will generalize to other types of rewards and punishments. Finally, in order to further evaluate whether stress-induced reward deficits are a potential mechanism underlying the link between stress and depression, it will be imperative to run parallel experiments in MDD individuals. In spite of these limitations, the current study has substantial translational value and significant strengths, including the use of a well-controlled experimental procedure (threat-of-shock) to superimpose an acute stress manipulation on a primary task (the PSST).

## Conclusions

In sum, results from the current biologically informed analyses support *a priori* hypotheses and previous research findings (Bogdan and Pizzagalli, 2006; Pizzagalli et al., 2007; Bogdan et al., 2010) by demonstrating that stress-reactive individuals under stress exhibit reduced reward processing (i.e., reduced sensitivity to positive feedback, evident in an impaired ability to use this reward information to guide decision making in novel contexts) relative to individuals not under stress. These results are also in line with recent neuroimaging studies that have shown reduced activation in reward-related neural areas in response to stress inductions implemented immediately prior to reward processing tasks (Ossewaarde et al., 2011; Porcelli et al., 2012). Critically, findings from the current study extend this area of research by providing initial evidence that these stress-induced deficits appear to be reward-specific and not generalizable to punishment processing. Given that negative life stress often precedes depression onset (Kendler et al., 1999) and predicts clinical severity (Tennant, 2002), the current results also provide support for the possibility that stress-induced hedonic deficits may be a potential mechanism underlying the connection between negative stress and depressive episodes. In this way, such results are in line with conceptualizations of stress-induced anhedonia as a potential vulnerability factor for depression (Berghorst and Pizzagalli, 2010, for review). Although promising, it is important to emphasize that (1) these findings emerged in the context of an only partially successful stress manipulation (see Appendix); (2) findings emerged only after a subgroup of stress-reactive participants was identified; and (3) the ecological validity of the stress manipulation was limited. Accordingly, these findings await replications and conclusions should be tempered. Future studies also need to examine whether the stress-induced rapid activation of the mesocortical DA system and inhibition of the mesolimbic DA system in animal models (Cabib and Puglisi-Allegra, 1996; Cabib et al., 2002) represent biological mechanisms fundamental to the current study findings.

### Conflict of interest statement

The authors declare that the research was conducted in the absence of any commercial or financial relationships that could be construed as a potential conflict of interest.

## Appendix

### Description of measures

#### Trait and dispositional self-report measures

The Beck Depression Inventory-II (BDI-II; Beck et al., 1996) is a 21-item questionnaire used to measure depressive symptoms over the past 2 weeks. It has strong internal reliability (0.86–0.92), high test-retest reliability over 1-week (0.93), and good convergent and discriminant validity (Beck et al., 1996; Steer et al., 2000; Segal et al., 2008).

The Mood and Anxiety Symptom Questionnaire (MASQ-short) is a 62-item questionnaire used to assess symptoms of anxiety and depression over the past week with good convergent and discriminant validity in clinical and community samples (Watson et al., 1995); it yields four subscales—general distress anxious, anxious arousal, general distress depressive, and anhedonic depressive.

The Perceived Stress Scale (PSS; Cohen et al., 1983) is a 14-item measure used to assess the degree to which an individual appraises the situations in his or her life as stressful over the past month. Internal reliability coefficients for the PSS range from 0.84 to 0.86 with a test-retest reliability of 0.85 (over 2 days); the measure has been demonstrated to have strong convergent validity (Cohen et al., 1983).

The Temporal Experience of Pleasure Scale (TEPS; Gard et al., 2006) is a 14-item measure used to assess individual trait dispositions in anticipatory and consummatory experiences of pleasure. The scale has good internal consistency (0.71–0.79), high test-retest reliability over 5–7 weeks (0.75–0.81), and strong convergent and discriminant validity (Gard et al., 2006).

The Behavioral Inhibition and Behavioral Activation Scales (BIS/BAS; Carver and White, 1994) are used to measure individual differences in sensitivity to two motivational systems purported to underlie behavior: a behavioral activation system and a behavioral inhibition system. It has good convergent and discriminant validity in community and clinical samples (Carver and White, 1994; Campbell-Sills et al., 2004).

#### “In-the-moment” state self-report measures

The state form of the State Trait Anxiety Inventory (STAI-S) includes 20 items used to quantify state anxiety levels. Internal consistency coefficients range from 0.86 to 0.95, while test-retest reliability coefficients (over 2 months) range from 0.65 to 0.75 (Spielberger et al., 1983).

The state version of the Positive and Negative Affect Schedule (PANAS) is used to measure current levels of positive and negative affect. Internal consistency coefficients range from 0.86–0.90 for the positive affect scale and 0.84–0.87 for the negative affect scale; test-retest reliability coefficients (over 2 months) range from 0.47–0.68 for the positive affect scale and 0.39–0.71 for the negative affect scale (Watson et al., 1988).

The Challenge-Threat Questionnaire (Mendes et al., 2001) was designed to assess individuals' threat appraisals (perceived resources/demands) of a task, with pre-task and post-task versions. Unfortunately, only 23 “controllable stress” participants and 21 “uncontrollable stress” participants completed this measure since it was added midway through data collection. The pre-task version typically includes 11 statements (e.g., “The upcoming task will take a lot of effort to complete,” “I have the abilities to perform the upcoming task successfully”) that participants rate on a scale from 1 (“strongly disagree”) to 7 (“strongly agree”) to indicate how they are feeling about the task they are about to complete. The pre-task version used in this study included two additional items to assess participants' perceived control over general task performance, and perceived control over whether shocks would occur in the upcoming task. Participants completed the pre-task form after receiving PSST instructions but prior to beginning the PSST. The post-task version typically includes nine statements (e.g., “The task was very demanding,” “I felt that I had the abilities to perform well in the task”), which participants again rate on a scale from 1 (“strongly disagree”) to 7 (“strongly agree”) to indicate how they feel about the task they just completed. The post-task version used in this study also included two additional items to assess participants' perceived control over general task performance, and perceived control over whether shocks occurred in the task. Participants completed the post-task form after finishing the PSST.

### Analyses

All analyses parallel those reported in the main manuscript (*Trait and dispositional self-report measures; “In-the-moment” state self-report measures; PSST training phase; PSST test phase*) except they were computed using *Group* with three levels (“no stress,” “controllable stress,” “uncontrollable stress”) in mixed ANOVAs.

### Results

#### Trait and dispositional self-report measures

There were no significant differences between groups on trait and dispositional self-report measures collected at baseline (all *Fs < 2.09*, *p*s > 0.13); see Table A1.

**Table A1:** **Demographics, trait and dispositional self-report measures of the original three groups**.

	**No stress group (*n*** = 27)	**Controllable stress group (*n*** = 34)	**Uncontrollable stress group (*n*** = 34)	**Statistics**	***p***
Gender (% female)	100%	100%	100%	N/A	N/A
Age (years)	21.43 (±1.79)	21.33 (±2.24)	21.32 (±2.20)	*F*_(2, 94)_ = 0.02	0.98
Education (years)	14.81 (±1.39)	14.44 (±1.69)	14.26 (±1.54)	*F*_(2, 94)_ = 0.96	0.39
Marital status (%single)	100%	91%	94%	χ^2^(1) = 5.37	0.25
Income (% <$50,000)	90%	73%	74%	χ^2^(1) = 2.29	0.32
Compensation form (% monetary)	85%	91%	88%	χ^2^(1) = 0.53	0.77
Ethnicity (% Hispanic)	7%	9%	6%	χ^2^(1) = 0.22	0.90
Ethnicity (% Caucasian)	85%	44%	74%	χ^2^(1) = 12.60	<0.01
BDI-II Score	1.85 (±2.38)	2.41 (±2.52)	2.00 (±2.16)	*F*_(2, 94)_ = 0.48	0.62
MASQ: GDA	15.52 (±4.74)	15.50 (±3.78)	15.82 (±4.06)	*F*_(2, 94)_ = 0.06	0.94
MASQ: GDD	16.85 (±5.25)	18.79 (±5.59)	17.41 (±4.59)	*F*_(2, 94)_ = 1.18	0.31
MASQ: AA	20.52 (±4.82)	19.94 (±4.32)	19.24 (±2.76)	*F*_(2, 94)_ = 0.79	0.46
MASQ: AD	49.56 (±10.90)	50.15 (±10.15)	49.26 (±11.32)	*F*_(2, 94)_ = 0.06	0.94
Perceived stress scale	19.67 (±6.33)	21.65 (±5.12)	19.71 (±6.45)	*F*_(2, 94)_ = 1.18	0.31
TEPS: anticipatory	64.67 (±6.68)	65.12 (±10.20)	64.18 (±9.46)	*F*_(2, 94)_ = 0.09	0.91
TEPS: consummatory	48.41 (±5.56)	50.82 (±6.04)	50.50 (±6.17)	*F*_(2, 94)_ = 1.41	0.25
BIS/BAS: reward responsiveness	7.48 (±1.67)	7.65 (±2.71)	7.38 (±1.50)	*F*_(2, 94)_ = 0.14	0.87
BIS/BAS: drive	9.19 (±1.96)	8.91 (±2.14)	9.21 (±2.14)	*F*_(2, 94)_ = 0.20	0.82
BIS/BAS: fun seeking	8.04 (±2.16)	7.82 (±2.36)	7.74 (±2.12)	*F*_(2, 94)_ = 0.14	0.87
BIS/BAS: inhibition	16.00 (±2.82)	15.15 (±2.81)	15.65 (±2.87)	*F*_(2, 94)_ = 0.70	0.50

BDI-II, Beck Depression Inventory-II; MASQ, Mood and Anxiety Symptom Questionnaire; GDA, General Distress Anxious; GDD, General Distress Depressive; AA, Anxious Arousal; AD, Anhedonic Depression; TEPS, Temporal Experience of Pleasure Scale; BIS/BAS, Behavioral Inhibition and Behavioral Activation Scales.

#### “In-the-moment” state self-report measures

***State anxiety***. The mixed ANOVA on STAI-S scores revealed a significant main effect of *Time* [*F*_(1, 92)_ = 65.68, *p* < 0.01] and, more critically, a *Time × Group* interaction [*F*_(2, 92)_ = 4.72, *p* = 0.01]; *Group* was not significant [*F*_(2, 92)_ = 2.71, *p* = 0.07]. Paired *t*-tests indicated that anxiety increased from baseline to PSST in the “controllable stress” group [*t*_(33)_ = 5.72, *p* < 0.01], the “uncontrollable stress” group [*t*_(33)_ = 6.29, *p* < 0.01], and the “no stress” group [*t*_(26)_ = 2.17, *p* = 0.04]. At baseline, there were no group differences [*F*_(2, 94)_ = 0.22, *p* = 0.81]. In line with hypotheses, anxiety levels during the PSST were significantly different between groups [*F*_(2, 94)_ = 5.04, *p* < 0.01]. Follow-up *t*-tests revealed that participants in both the “controllable stress” [*t*_(59)_ = 2.67, *p* = 0.01] and uncontrollable stress groups [*t*_(59)_ = 3.00, *p* < 0.01] reported significantly higher anxiety than participants in the “no-stress” group. However, contrary to hypotheses, participants in the “controllable stress” group did not differ from those in the “uncontrollable stress” group [*t*_(66)_ = −0.24, *p* = 0.81].

***State negative affect***. The mixed ANOVA on PANAS-NA scores also revealed a significant main effect of *Time* [*F*_(1, 92)_ = 16.87, *p* < 0.01] and a *Time × Group* interaction [*F*_(2, 92)_ = 3.29, *p* = 0.04]; *Group* was not significant [*F*_(2, 92)_ = 2.55, *p* = 0.08]. Paired *t*-tests indicated that negative affect increased significantly from baseline to PSST in the “controllable stress” group [*t*_(33)_ = 2.76, *p* < 0.01] and the “uncontrollable stress” group [*t*_(33)_ = 3.50, *p* < 0.01], but not in the “no stress” group [*t*_(26)_ = 0.62, *p* = 0.54]. At baseline, there were no group differences in negative affect [*F*_(2, 94)_ = 0.25, *p* = 0.78]. However, negative affect during the PSST was significantly different between groups [*F*_(2, 94)_ = 3.52, *p* = 0.03]. Follow-up *t*-tests revealed that participants in both the “controllable stress” [*t*_(59)_ = 2.02, *p* < 0.05] and “uncontrollable stress” [*t*_(59)_ = 2.61, *p* = 0.01] groups reported significantly higher negative affect than participants in the “no-stress” group. However, again contrary to hypotheses, the two stress groups did not differ in their levels of negative affect during the PSST [*t*_(66)_ = −0.85, *p* = 0.40].

***State positive affect***. The mixed ANOVA on PANAS-PA scores revealed a main effect of *Time* [*F*_(1, 92)_ = 18.37, *p* < 0.01]; the *Time × Group* interaction [*F*_(2, 92)_ = 1.50, *p* = 0.23] and the *Group* main effect [*F*_(2, 92)_ = 1.05, *p* = 0.36] were not significant. All participants reported a reduction in positive affect from baseline to PSST.

***Challenge-threat questionnaire***. Contrary to hypotheses, the “controllable stress” and “uncontrollable stress” groups were not significantly different in their pre-task [*t*_(42)_ = 0.37, *p* = 0.71] or post-task [*t*_(42)_ = 0.28, *p* = 0.78] threat appraisals. Moreover, the two stress groups did not differ in their ratings of control over performance in the task prior to task onset [*t*_(42)_ = −0.03, *p* = 0.98] or after completing the task [*t*_(42)_ = 0.33, *p* = 0.74]. In both groups and at both assessments, these ratings were close to “neutral” but fell on the “disagree” side of the scale (<4) with regard to having control over their performance.

A mixed ANOVA on ratings of perceived control over shock with *Group* (Uncontrollable Stress, Controllable Stress) as a between-subjects variable and *Time* (Pre-PSST, Post-PSST) as a within-subjects variable revealed a trend for a *Time × Group* interaction [*F*_(1, 42)_ = 3.42, *p* = 0.07], with significant main effects of *Time* [*F*_(1, 42)_ = 29.60, *p* < 0.01] and *Group* [*F*_(1, 42)_ = 45.64, *p* < 0.01]. On pre-task ratings of control over shock, the “controllable stress” group was significantly higher than the “uncontrollable stress” group [*t*_(42)_ = 5.66, *p* < 0.01], as predicted; however, importantly and contrary to expectations, both groups again fell in the “disagree” zone of the rating scale (<4). A paired *t*-test within the “controllable stress” group indicated that they reported significantly more control over the shock at their post-task than pre-task rating [mean increased to 5.39 ± 1.62; *t*_(22)_ = 5.51, *p* < 0.01]; interestingly, the “uncontrollable stress” group also had a significant increase in level of perceived control over shock from pre-task to post-task [2.43 ± 1.75; *t*_(20)_ = 2.38, *p* = 0.03].

Overall, findings from the state measures indicated that the “threat-of-shock” stress manipulation induced significantly higher levels of negative affect and anxiety in both stress conditions than the no-stress condition, but no significant differences between the two stress groups. Further indications that the stress manipulation was only partially successful include the following: no significant differences between the two stress groups on pre-task threat appraisals or perceived control over general task performance, and pre-task ratings of control over shock were in the “disagree” zone of the scale for both groups.

#### Cortisol levels

The *Time* (T1 = baseline, T2 = post-“filler”-task/pre-PSST, T3 = post-PSST) × *Group* ANCOVA on cortisol levels, with “time since waking” as a covariate, revealed only a significant main effect of *Time* [*F*_(2, 176)_ = 11.37, *p* < 0.01]. Consistent with cortisol's diurnal pattern, cortisol levels dropped throughout the experiment [linear effect: *F*_(1, 88)_ = 15.14, *p* < 0.01]. Similarly, an ANOVA comparing groups on cortisol reactivity scores at T2-T1, and a separate ANOVA comparing groups on cortisol reactivity scores at T3-T1, yielded insignificant findings (all *F* < 1.78, *p* > 0.17). The unpaired *t*-test comparing the “controllable stress” group with the “uncontrollable stress” group on cortisol reactivity scores at T3-T1 was not significant [*t*_(64)_ = 0.36, *p* = 0.72], suggesting that both stress conditions yielded physiologically similar responses.

#### PSST training phase

Groups did not differ in the number of completed training blocks [*F*_(2, 94)_ = 0.49, *p* = 0.61]; all groups took approximately three blocks to advance to the test phase (no-stress group: 3.15 ± 1.75; controllable stress group: 3.06 ± 1.50; uncontrollable stress group: 3.44 ± 1.73). In the ANOVA for accuracy on the final training block with *Trial Type* (AB, CD, EF) and *Group* as factors, there was only a main effect of *Trial Type* [*F*_(2, 184)_ = 14.86, *p* < 0.01; all other *F*s < 1.30, *p*s > 0.30]; as expected, participants were most accurate on the AB trial type and least accurate on the EF trial type. No significant differences emerged from the ANOVA for RT on the final training block (all *F*s < 1.91, *p*s > 0.15). Altogether, these findings indicate that (1) the probabilistic contingencies elicited the intended behavioral effects, and (2) groups did not differ in performance during the training phase.

#### PSST test phase

The ANOVA comparing accuracy on AB trials (the “easiest” trial type) in the test phase with *Group* confirmed that there were no significant group differences in terms of participants learning the basic task [*F*_(2, 94)_ = 0.62, *p* = 0.54]. For accuracy, contrary to hypotheses, the *Trial Type* (“Choose A,” “Avoid B”) × *Group* ANOVA revealed no significant effects (all *F*s < 1.59, *p*s > 0.21).

For RT scores, the analogous *Trial Type* × *Group* ANOVA yielded a significant main effect of *Trial Type* [*F*_(1, 92)_ = 29.73, *p* < 0.01] and a *Trial Type × Group* interaction [*F*_(1, 92)_ = 4.56, *p* = 0.01]. Follow-up analyses indicated no significant group differences on “Choose-A” trials or “Avoid B” trials (all *p*s > 0.058). Paired *t*-tests revealed that participants in the “no stress” and “uncontrollable stress” groups were slower on their “Avoid B” trials than their “Choose A” trials [no-stress group: *t*_(26)_ = 4.47, *p* < 0.01; uncontrollable stress group: *t*_(33)_ = 4.49, *p* < 0.01]. Participants in the “controllable stress” condition, however, exhibited RTs that were not significantly different across trial types [*t*_(33)_ = 0.72, *p* = 0.48].

### Discussion

Inspired by non-human animal research documenting that uncontrollable stressors may be potent triggers of anhedonic-like behavior, we attempted to examine whether stressor controllability moderates the relationship between stress and reward processing dysfunction. Although the stress manipulation did induce significantly higher levels of negative affect and anxiety than the no-stress condition, the uncontrollable and controllable stress manipulations elicited similar affective and cortisol responses, which was contrary to hypotheses. Notably, these results echoed patterns with self-report measures indicating that the “controllable stress” group did not actually believe they had control over the stressor. Accordingly, due to an only partially successful stress manipulation, conclusions could not be drawn concerning the impact of perceived control over stress.

Contrary to expectations, the two stress groups (“controllable” and “uncontrollable”) did not differ significantly from each other in their levels of anxiety or negative affect. Cortisol reactivity analyses similarly did not reveal differences between the “controllable stress” and “uncontrollable stress” groups. Moreover, there were no significant differences between the two stress groups on pre-task threat appraisals (perceived demands/personal resources) or perceived control over general task performance. Although pre-task ratings of control over shock were higher in the “controllable stress” group than the “uncontrollable stress” group, both groups' ratings fell in the “disagree” zone of the scale, indicating that prior to task onset, subjects in the “controllable stress” group did not actually believe that they would have control over the stressor. This lack of believability may stem from the fact that participants in the “controllable stress” group were told they would be able to “significantly reduce” the likelihood of receiving shock by pressing down on the foot pedal, but could not completely eliminate the possibility of being shocked (i.e., they were not given “complete” control). Task instructions were outlined this way because of concerns that the latter set of instructions would not induce significantly more stress than the no-stress condition. Collectively, these data suggest that the stress manipulation was only partially successful: significantly more negative affect and anxiety was reported by participants in both stress groups relative to the “no-stress” group, but the controllability manipulation was not successful.

Results from this aspect of the experiment serve to highlight key variables to consider in the design of future experiments. For example, the importance of administering an assessment of perceived control over stress *prior* to task onset and collecting data on a physiological index of stress (e.g., cortisol levels) to confirm the effects of any stress manipulation on participants. Moreover, given that participants in our “controllable” stress condition (who were told they had “partial” control over the stressor) did not report truly believing they had control over the stressor, future designs warrant including a “controllable stress” condition in which participants are given perceived *full* control over the stressor.
